# A NEW INSTRUMENT TO DETERMINE ADEQUATE STAFFING RATIOS IN GERMAN NURSING HOMES

**DOI:** 10.1093/geroni/igz038.1398

**Published:** 2019-11-08

**Authors:** Karin Wolf-Ostermann, Heinz Rothgang, Ingrid Darmann-Fink, Thomas Kalwitzki, Mathias Fünfstück

**Affiliations:** University of Bremen, Bremen, Bremen, Germany

## Abstract

Ever since mandatory long-term care insurance was introduced in Germany there has been concern about staffing of nursing homes. As attempts to introduce the Canadian PLAISIR system failed today staffing ratios between federal states differ by more than 20 % and perceived understaffing is a major reason for the lack of nurses. Against this background the reform act from December 2015 commissioned the development of a new instrument to identify necessary staffing ratios. The University of Bremen was mandated to develop this instrument, and will present the final product in August 2019 to the Commissioner. The contribution will describe the methods applied in developing the instrument and the resulting instrument itself which translates the number and characteristics of any given nursing home into staffing requirements according to certain degrees of qualification, thus replacing general quotas of registered nurses to auxiliary staff by individual ratios for each nursing home.

